# New Insight into microRNA Functions in Cancer: Oncogene–microRNA–Tumor Suppressor Gene Network

**DOI:** 10.3389/fmolb.2017.00046

**Published:** 2017-07-07

**Authors:** Kecheng Zhou, Minxia Liu, Yi Cao

**Affiliations:** ^1^Laboratory of Molecular and Experimental Pathology, Kunming Institute of Zoology, Chinese Academy of SciencesKunming, China; ^2^Kunming College of Life Science, University of Chinese Academy of SciencesKunming, China

**Keywords:** oncogene–microRNA–tumor suppressor gene network, miRNA function, cancer, oncogenes, tumor suppressor genes

## Abstract

Tumorigenesis is a multi-step and complex process with multi-factors involved. Deregulated oncogenes and tumor suppressor genes (TSGs) induced by genetic and epigenetic factors are considered as the driving force in the development and progression of cancer. Besides, microRNAs (miRNAs) act vital roles in tumorigenesis through regulating some oncogenes and TSGs. Interestingly, miRNAs are also regulated by oncogenes and TSGs. Considering the entangled regulation, here we propose a new insight into these regulation relationships in cancer: oncogene–miRNA–TSG network, which further emphasizes roles of miRNA, as well as highlights the network regulation among oncogene, miRNA, and TSG during tumorigenesis. The oncogene–miRNA–TSG network demonstrates that oncogenes and TSGs not only show functional synergy, but also there are regulatory relationships among oncogenes and TSGs during tumorigenesis, which could be mediated by miRNAs. In view of the oncogene–miRNA–TSG network involved in many oncogenes, miRNAs, and TSGs, as well as occurring in various tumor types, the anomaly of this network may be a common event in cancers and participates in tumorigenesis. This hypothesis broadens horizons of molecular mechanisms underlying tumorigenesis, and may provide a new promising venue for the prediction, diagnosis, and even therapy of cancer.

## Introduction

Tumorigenesis is a complicated process, induced by multi-factors, such as environment, genetics, and epigenetics. Mechanisms underlying tumorigenesis are involved in gene mutation, chromosome stability, DNA repair, epigenetic changes, and cell growth, differentiation, movement, apoptosis, autophagy, and so on. Abnormal expressions of oncogene and tumor suppressor gene (TSG) are regarded as the key driving force promoting the cell malignant transformation. For instance, loss of p53 (an important TSG) promotes cell proliferation (Drosten et al., 2014), and disturbs p53-dependent apoptosis (Vazquez et al., 2008); meanwhile, activation of p53 often occurs in the response of DNA damage (Lakin and Jackson, 1999; Smith and Seo, 2002); activated Myc (an important oncogene) was involved in dysfunction of several important cell processes, including growth control (Schmidt, 1999), apoptosis (Hoffman and Liebermann, 2008), and DNA damage response and repair (Campaner and Amati, 2012; Li et al., 2012).

MicroRNAs (miRNAs), a post-transcriptional level regulator, have fundamental activities in cancer development and progression by modulating oncogene and/or TSG through specifically targeting messenger RNA (mRNA) 3′-untranslated region (UTR), either degrading targeted mRNA or blocking translation. Numerous studies indicated that miRNAs act as oncogenic miRNA (by targeting TSG) or tumor suppressive miRNA (by targeting oncogene). Interestingly, some oncogenes or tumor suppressor genes (TSGs) are capable of activating miRNA transcription by binding to promoter regions of miRNA host genes. MiRNA expressions are also regulated by oncogene and/or TSG in addition to mutations and epigenetic changes (mainly methylation) of miRNA host genes. Therefore, miRNAs are involved in the construction of convoluted signal transduction pathways together with oncogene and TSG. Here, we propose oncogene–miRNA–TSG network hypothesis, which highlights miRNAs may function as mediators among oncogenes as well as TSGs, and as key nodes of the collaborated regulation network. Furthermore, the hypothesis provides other potential regulation relationships among oncogenes and TSGs.

## Functions and functional synergy of oncogene and TSG

**Figure 1:**
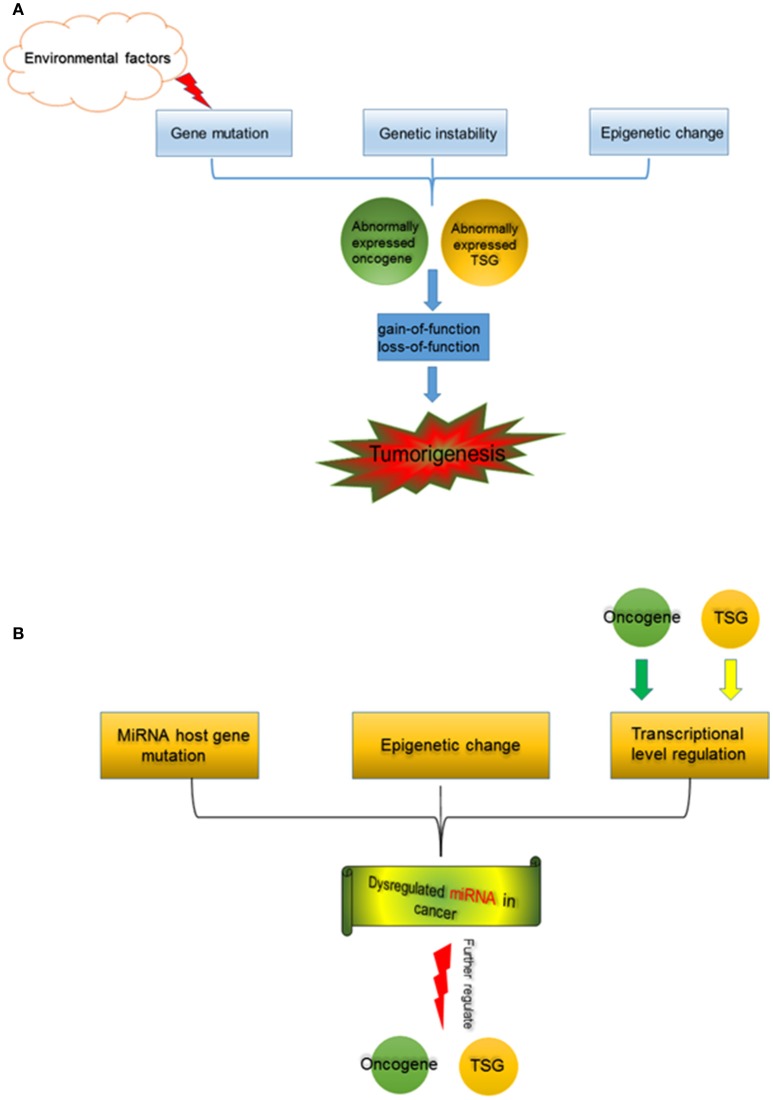
**(A)** Tumorigenesis is a multi-factor induced process. Gene mutation, which could be induced by environmental factors (such as chemical carcinogens, physical carcinogens, pathogenic bacteria, and viruses*)*, and genetic instability, as well as alterations in epigenetics cause abnormal expression of oncogene and TSG. Functional changes of oncogene and TSG, separately or jointly drive tumorigenesis. **(B)** miRNA in cancer. MiRNA host gene mutation, epigenetic change (including aberrant DNA demethylation, aberrant modification of histone deacetylase, etc.), and the transcription regulation by oncogene and TSG contribute to miRNA dysregulation in cancer. The abnormally expressed miRNAs further regulate oncogene and TSG during tumorigenesis.

Many factors are involved in tumorigenesis and promote the cell malignant transformation, consequences of the characters or the hallmarks of cancer (Hanahan and Weinberg, 2011). Functional alterations of oncogene and TSG are one of the most important mechanisms underlying tumorigenesis (Figure 1A). Gene mutation can induce functional changes of gene. Many environmental factors, for instance, chemical carcinogens (e.g., benzopyrene from smoking and air pollution; Hecht, 1999), physical exposure (such as ultraviolet irradiation and radiation exposure), as well as pathogenic bacteria and viruses (including human hepatitis B and C virus, and human papilloma virus), induce somatic mutation (Minamoto et al., 1999). Random mutations during DNA replication in stem cells are also an important source of somatic mutations (Tomasetti and Vogelstein, 2015; Tomasetti et al., 2017). Germline mutations arise from parent heredity. A sequence of oncogene and TSG mutations including somatic mutations and germline mutations could result in the cell malignant transformation (Luzzatto, 2011). Genetic instability and mutations are not only the hallmark of cancer, but also central to the genesis, development, and evolution of cancer (Loeb and Loeb, 2000). The gene mutation leads to either the active function, like EGFR in non-small cell lung cancer (NSCLC) (Lynch et al., 2004), or the inactive function, like p53 mutation (Muller and Vousden, 2013). The gain-of-function of oncogene and loss-of-function of TSG play key roles during tumorigenesis, and also provide potential therapeutic targets (Paez et al., 2004; Hong et al., 2014), even though current situation is still challenging (Yu et al., 2014).

Epigenetic changes, including DNA methylation (Worm et al., 2002), histone modifications (Cohen et al., 2011), chromatin remodeling (Wolffe, 2001), as well as miRNAs (Kala et al., 2013), etc. also induce dysfunction of oncogene and TSG, which are observed in kinds of cancers (Grønbæk et al., 2007; Singh et al., 2016; Wijetunga et al., 2016). Many studies showed that the collaboration of epigenetic and genetic changes is critically vital to drive the cancer development and progression (Baylin and Jones, 2016).

It has been noted that multi-factors and series cell processes emerge during tumorigenesis. By preforming whole-genome-sequencing studies of 3281 tumors from 12 cancer types, more than 600,000 somatic mutations were observed in cancer cells (Kandoth et al., 2013). Those mutations occur in plenty of oncogenes and TSGs, further induce either gain-of-function or loss-of-function. Eventually, altered oncogenes and TSGs collaboratively drive the cancer development and progression.

## MiRNA roles in cancer

MiRNA is a vital factor contributing to the epigenetic regulation. After its first discovery (Lee et al., 1993), miRNAs were quickly shown with a conserved mechanism and broad functional significance (Bartel, 2004). Moreover, abnormally expressed miRNAs are useful biomarkers for cancer diagnosis, and even promising targets for cancer therapy (Hayes et al., 2014).

Origins of dysregulated miRNAs are various in cancer cells (Figure 1B). MiRNA host gene codes miRNA, and it can regulate miRNA expression at the transcription level. Some mutations within miRNA host genes induced incorrect miRNA expressions at the DNA level. MiRNA dysregulation induced by gene mutation was observed in cancers and other diseases (Meola et al., 2009). Interestingly, in colorectal cancers from Kashmiri population, mutations/SNPs within miRNA genes or their binding sites in 3′-UTR are infrequent events, indicating that miRNA gene mutations may not be as common as mutations of protein coding genes during tumorigenesis (Maqbool et al., 2014). Epigenetic changes are also vital factors contributing abnormal miRNA expressions in cancer. A research revealed that the 17 miRNAs were up-regulated by simultaneous treatment with the chromatin-modifying drugs 5-aza-2′-deoxycytidine (5-Aaza-Ccdr; a DNA methyltransferase inhibitor) and 4-phenylbutyric acid (PBA; a histone deacetylase inhibitor), demonstrating that epigenetic mechanisms (DNA demethylation and histone acetylation) could alter miRNA expressions (Saito et al., 2006). In addition, 5-Aaza-Ccdr and PBA treatments activated the miR-512-5p expression in gastric cancer cells, which further caused the MCL1 suppression and resulted in the cell apoptosis (Saito et al., 2009). These confirmed that chromatin remodeling induced by the epigenetic treatment was capable to directly affect specific miRNA expression.

Apart from genetic and epigenetic mechanisms, transcription factors can also bind to promoter regions of miRNA host genes, and regulate miRNA expression. Transcription factor binding sites were highly enriched within miRNA precursor sequences, suggesting that transcription factors may directly bind to primary miRNA gene transcripts or small hairpin miRNA precursors, thus, regulate their processing (Piriyapongsa et al., 2011). Moreover, some well-known oncogenes and TSGs regulate miRNA expression at the transcription level. For instance, c-Myc up-regulated miR-17 ~ 92 and miR-106a ~ 363 expressions in cultured human trophoblasts (Kumar et al., 2013). Jackstadt et al carried out genome-wide analyses of c-Myc-regulated mRNAs and miRNAs, collaborating an accelerated and comprehensive understanding of c-Myc function (Jackstadt et al., 2013). Strikingly, c-Myc also transactivated drosha expression though directly binding to the E-box of the drosha promoter (Wang et al., 2013), which imposed on board and general regulation of miRNAs. P53 could activate miR-34 family, such as miR-34a (Chang et al., 2007), miR-34b, and miR-34c (Corney et al., 2007), further affected cell apoptosis, proliferation, and adhesion-independent growth.

Interestingly, oncogene and TSG are also targets of miRNAs (Figure 1B). MiRNA let-7a down-regulated Myc, and thus reverted the Myc-Induced cell growth in burkitt lymphoma cells (Sampson et al., 2007); miR-34a modulated c-Myc transcriptional complexes, suppressing malignancy in human prostate cancer cells (Yamamura et al., 2012). MiR-504 acted as a negative regulator of p53 by directly binding to the 3′-UTR, therefore, decreased the p53 mediated-apoptosis and cell-cycle stress (Hu et al., 2010). Additionally, miR-125b was a novel regulator of p53, further affects the p53-induced apoptosis during development and stress response (Le et al., 2009). Besides c-Myc and p53, other oncogenes and TSGs were regulated by miRNAs as well, and involved in the development and progression of cancer. For instance, miR-144 targeted oncogene ZEB1, and a decreased miR-144 expression resulted in the increased Zeb1 expression and epithelial-mesenchymal transition (EMT) (Pan et al., 2015). Another study identified three differentially expressed miRNA in metastases as key drivers of EMT though targeting SIAH1, SETD2, ZEB2, and FOXN3 (Mudduluru et al., 2015). Currently, we found miR-203a regulated the expression of ERGIC3 (a candidate oncogene), and further affected cell proliferation in lung cancer (Lin et al., 2015).

It is worthy to note about several characters of miRNA regulation and related problems in miRNA researches. (1) MiRNA regulation is a dynamic process. By using Affymetrix microarray platform, Kumari et al profiled miRNA and mRNA expressions at multiple time points, and revealed the dynamic changes in global miRNAome and transcriptome (Kumari et al., 2016). A previous study also suggested the dynamic modeling of miRNA regulation during the mesenchymal stem cell differentiation (Weber et al., 2013). Thus, the dynamic character implies that miRNA study needs to consider the effects of time and state on results. (2) One miRNA can target multiple mRNAs, thence, one miRNA has different functions. For example, miR-21 affected cell survival by targeting PCPD4 (programmed cell death protein 4) (Jiao et al., 2015), regulated cell proliferation by targeting PTEN (Zhang et al., 2010), and meanwhile was involved in cancer metastasis by targeting Maspin (Zhu et al., 2008). Sometimes, opposite functions were observed for one miRNA in diverse cases, because it targeted different mRNAs in specific cell types or cell states. For instance, one study suggested miR-944 functioned as a novel oncogenic miRNA and regulated the chemoresistance in breast cancer (He et al., 2016), meanwhile another study reported that the miR-944 expression was severely repressed in breast cancer cells and clinical specimens, and suppressed the cell migration by targeting SIAH1 (Flores-Pérez et al., 2016). Interestingly, our recent study revealed miR-944 was significantly down-regulated in NSCLC by utilizing small RNA deep sequencing, and found that miR-944 inhibited cellular proliferation through targeting EPHA7 in NSCLC, which may offer a new mechanism underlying the development and progression of NSCLC (Liu et al., 2016). Therefore, when studying miRNA functions, it is necessary to declare detail mechanisms and targets. Since one specific miRNA could possess various functions, physiological roles of miRNA should be further investigated by animal models, such as knockout and transgenic animals. (3) One mRNA is regulated by multiple miRNAs simultaneously, which may be involved in the complexity of miRNA synergy and may be one reason of practical problems in miRNA studies. Utilizing miRNA mimic and miRNA inhibitor to study the miRNA's target and examine its related function is a classical and established strategy. However, in practice, effects of miRNA mimic and inhibitor are not always at opposite ends, some discrepancies results by using miRNA mimics and antisense inhibitors were observed (Thomson et al., 2013). Occasionally, transfected small RNA may compete with endogenous miRNA for the RNA-induced silencing complex (RISC) or other machinery, thus, could result in artificial readout (Khan et al., 2009). Meanwhile, miRNA inhibitors may not be sufficiently specific to separate different members from the same miRNA family harboring similar sequences (Thomson et al., 2011). Another potential explanation may be due to neutralization and compensation mechanisms. Briefly, after one miRNA is modulated through mimic or inhibitor treatments, other miRNAs which target the same mRNA like this miRNA, may change their expressions to neutralize or compensate the biological effects.

## Oncogene–miRNA–TSG network hypothesis

**Figure 2:**
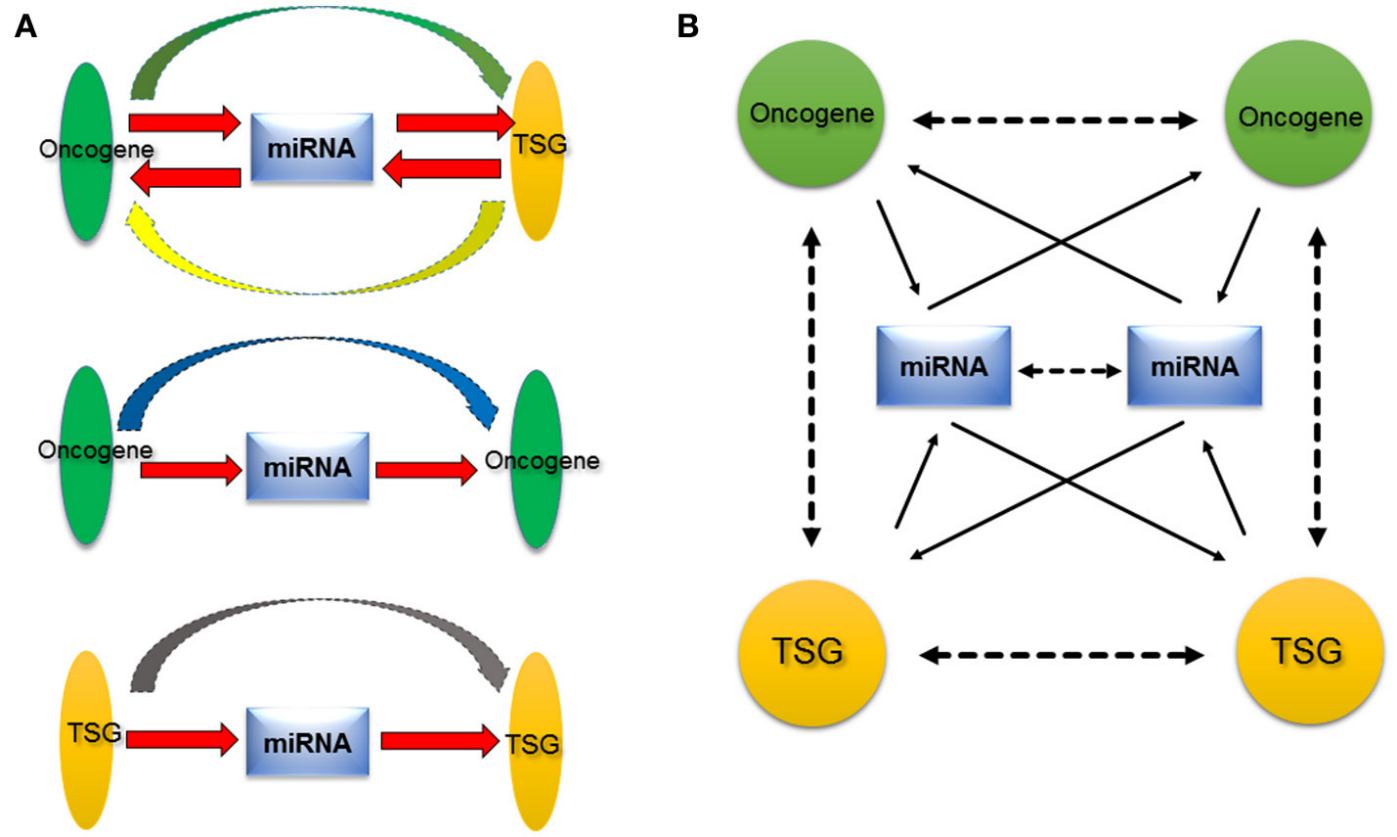
Proposed oncogene-miRNA-TSG regulation network. **(A)** MiRNA acts a central role among oncogene or TSG. On one hand, miRNA regulates oncogene or TSG, on the other hand, miRNA is regulated by oncogene or TSG (red arrow). Therefore, miRNA may serve as transductor/mediator among oncogene and TSG. Besides the functional synergy of oncogene and TSG, there are regulatory relationships between oncogene and TSG (green and yellow arrow), between oncogenes (blue arrow), and between TSGs (gray arrow). **(B)** Because the existence of numerous oncogenes, miRNAs, and TSGs, their relationships are presented as a complex network, the oncogene–miRNA–TSG network. The network provides a new insight into roles of miRNAs, oncogene, and TSG in cancer. Solid arrows indicate direct relationships that have been verified with experiments at the molecular level, and dotted arrows denote indirect relationships that are supported by experiments at the cellular level.

In early studies, we screened novel cancer-related genes and found that microspherule protein 1 (MCRS1) was overexpressed in lung cancer (Liang et al., 2013). Subsequently, we confirmed the overexpression of MCRS1 was associated with the cellular proliferation, EMT, and metastasis (Liu et al., 2014). MCRS1 was a candidate oncogene. Furthermore, MCRS1 transactivated the miR-155 expression by directly binding to its promoter region (Liu et al., 2014). Interestingly, in the continuing study, we established that MCRS1 promoted cancer cell growth via miR-155 targeting Rb1 (Liu et al., 2015). Thus, there is the MCRS1–miR-155–Rb1 pathway in lung cancer. MiR-155 mediated the regulation relationship between MCRS1 (a candidate oncogene) and Rb1 (a famous TSG), suggesting miRNAs could potentially act as a mediator between oncogene and TSG. Additionally, a recent study demonstrated that miR-200 mediated the regulation relationship between p53 (a well-established TSG) and ZEB1/BMI1 (a well-known oncogene), and p53–miR-200–ZEB1/BMI1 contributed to EMT in breast cancer cells (Chang et al., 2011). Meanwhile, MCRS1 and Rb1, p53 and ZEB1/BMI1 are simultaneously regulated by other miRNAs, such as miR-129^*^ (targeting MCRS1) and miR-125b (targeting p53), respectively. Taking those regulation pathways into consideration, we thought that oncogenes/TSGs can regulate miRNAs expression by binding to promoter regions; and the disrupted miRNAs, in turn, continue to regulate other oncogenes/TSGs. Here, we propose a new hypothesis, oncogene–miRNAs–TSG network (Figure 2). MiRNAs act as transductor/mediator among oncogenes and/or TSGs. In one case, miRNAs regulated by oncogenes further target TSGs, to collaborate and accelerate effects of oncogene (the miRNA upstream). In another case, if the miRNA regulated by one oncogene further aims at another oncogene, this oncogene 1–miRNA–oncogene 2 feedback will alleviate the effect of oncogene 1, suggesting that there may be precise regulation relationships among oncogenes. For examples, oncogenic KRAS regulated miR-200c (Tsunoda et al., 2011), while miR-200c targeted ZEB1/ZEB2 (Korpal et al., 2008) (ZEB1/ZEB2 functions as an oncogene to promote the cancer metastasis). Interestingly, miR-200c was also shown to target KRAS (Kopp et al., 2014). Here, miR-200c mediated the regulation relationship among oncogenes (KRAS, ZEB1/ZEB2), and acted the feedback regulation of one oncogene (KRAS). Considering the regulation relationships involved in many oncogenes, miRNAs, and TSGs, we extend the hypothesis to oncogene/TSG–miRNA–oncogene/TSG network (in order to express the convenience and simplicity, we still used “oncogene–miRNA–TSG network” in this article), which addresses the function of miRNA, and the complexity of the regulation network as well.

We think that this oncogene–miRNA–TSG network may be prevalent in normal cells too, and it generates various regulation relationships and possesses important biological functions, which is extremely essential to keep intermolecular homeostasis for the normal status of cells. In diseases, particularly in cancers, dysregulations of certain oncogenes, miRNAs, and TSGs that were induced by genetic mutations or epigenetic changes *etc.*, disrupt the balance of this molecular network, and transduct mislead signals to more downstream pathway molecules (including oncogene, miRNA, and TSG), and arouse domino effects, therefore, promote the cell malignant transformation. The oncogene–miRNA–TSG network is involved in many oncogenes, miRNAs, and TSGs, as well as occurs in various tumor types, hence it may participate in the development and progression of a wide range of tumors. The anomaly of this network may be a common event in cancers. In addition, the oncogene–miRNA–TSG network hypothesis demonstrated that oncogenes and TSGs not only show functional synergy, but also there are regulatory relationships among oncogenes and TSGs during tumorigenesis, which could be mediated by miRNAs. Finally, this hypothesis also addresses that tumorigenesis may be a result of the loss of the intermolecular homeostasis, scilicet, the disruption of entire oncogene–miRNA–TSG network rather than single dysregulation of oncogene or TSG. MiRNAs can be released through exosome from cancer cells into body fluids, such as blood, urine, milk, sputum, saliva. These miRNAs can serve as biomarkers for diagnosis (Tran, 2016). Recognizing the significances of oncogene–miRNA–TSG network may help better understanding oncogenes, TSGs, and miRNAs roles during the development and progression of cancer, and may provide a new promising venue of prediction, diagnosis, and therapy of cancer.

## Author contributions

KZ, ML, and YC jointly wrote this review. KZ drafted the manuscript. ML participated in in-depth discussions and made constructive suggestions and comments. YC developed the structure, design the scope of the review, and helped to draft the manuscript. All authors read and approved the final manuscript.

### Conflict of interest statement

The authors declare that the research was conducted in the absence of any commercial or financial relationships that could be construed as a potential conflict of interest.
